# Assessment of sexual function and its association with quality of life and disease severity in patients with atopic dermatitis^☆^

**DOI:** 10.1016/j.abd.2025.501259

**Published:** 2026-01-07

**Authors:** Aline Bressan, Natalia Troncoso, Carla Jorge Machado, Rita Fernanda Cortez de Almeida, Sueli Carneiro

**Affiliations:** a Department of Dermatology, Universidade do Estado do Rio de Janeiro, Rio de Janeiro, RJ, Brazil; b Department of Preventive and Social Medicine, Universidade Federal de Minas Gerais, Belo Horizonte, MG, Brazil

Dear Editor,

Atopic Dermatitis (AD) is a chronic inflammatory and pruritic condition that affects up to 25% of children and between 1% and 10% of adults, with no gender preference.^1^ Although most patients experience resolution of symptoms in adulthood, between 10% and 30% continue to exhibit the condition, and a smaller portion develops the initial symptoms in adulthood.^2^ This condition significantly impacts patients’ quality of life, yet its effects on sexual function remain underexplored.^3^

We report preliminary findings from a cross-sectional study examining sexual function and health-related quality of life in patients with AD.

Between January 2022 and January 2024, we evaluated 70 patients with AD (40 women and 30 men; mean age 32.9 ± 13.6 years) at our university hospital’s dermatology clinic. Eligible participants were aged 18 to 79 years, had a confirmed diagnosis of AD based on Hanifin-Rajka criteria, were sexually active, and literate.^1^ Patients with chronic debilitating illnesses were excluded. Disease severity was assessed using the SCORAD index.^4^ Sexual function was measured by the Female Sexual Quotient (QS-F) and the Male Sexual Quotient (QS-M) .^5^, ^6^ Health-related quality of life was evaluated using the EQ-5D-3L, a standardized instrument that measures perceived health status across five dimensions: mobility, self-care, usual activities, pain/discomfort, and anxiety/depression. Responses are converted into a utility index ranging from 0 (equivalent to death) to 1 (perfect health).^7^, ^8^ Statistical analysis included Student’s *t*-test, proportions’ exact test comparisons (Fisher test), and Spearman’s correlation analyses, with significance set at p < 0.05. In addition to significance testing, statistical power was estimated for each comparison to assess the reliability of the observed differences and to account for the probability of type II error (ability to identify a real significant difference). For Spearman’s correlation coefficient, approximate power was calculated by converting the observed coefficient to its Pearson-equivalent.

Patients had a mean age of 32.9 ± 13.6 years and an average disease duration of 18.8 ± 11.0 years (Table 1). There were no significant differences between men and women in these parameters (power < 0.20 for both comparisons). The low power suggests these analyses had limited ability to detect meaningful differences if they existed. The mean SCORAD score was 29.7 ± 19.3, with 42.9% of patients presenting moderate disease and 41.4% classified as mild, again with no gender-based differences (power < 0.20). Again, the insufficient power limits confidence in ruling out clinically relevant differences. The average EQ-5D-3L index was 0.68 ± 0.20, with no significant differences between sexes and low power (below 0.20) (Table 1). Our overall EQ-5D-3L was significantly lower than Brazilian population norms (0.82 ± 0.17, p < 0.001, power = 0.99), indicating substantial impairment in perceived health status,^9^ with a very high probability of identifying a significant difference (high statistical power). The most compromised dimensions were pain/discomfort, reported by 88.6% of participants, and anxiety/depression, reported by 82.9%.

**Table 1:** Sociodemographic and clinical characteristics of the sample (n = 70).

Characteristic	Total (n = 70)	Women (n = 40)	Men (n = 30)	p-value (type I error)	Statistical power (1- type II error)
Age of the patient (years): mean ± SD	32.9 ± 13.6	34.5 ± 13.3	30.8 ± 13.8	0.260	0.204
Duration of disease (years): mean ± SD	18.8 ± 11.0	19.1 ± 11.7	18.5 ± 10.2	0.860	0.056
SCORAD (continuous): mean ± SD	29.7 ± 19.3	31.6 ± 19.8	28.3 ± 19.0	0.475	0.108
SCORAD (categorical): n (%)					
- Mild (< 25)	29 (41.4)	12 (40.0)	17 (42.5)	0.851	0.027
- Moderate (25‒50)	30 (42.9)	14 (46.7)	16 (40.0)		0.049
- Severe (> 50)	11 (15.7)	4 (13.3)	7 (17.5)		0.037
EQ-5D-EL (continuous): mean ± SD	0.68 ± 0.20	0.65 ± 0.17	0.73 ± 0.23	0.061	0.361

SD, Standard Deviation; SCORAD, Scoring Atopic Dermatitis; EQ-5D-3 L, Health Questionnaire.

Regarding sexual function, 77.1% of participants reported being sexually active, with no significant difference between women (75.0%) and men (80.0%; p = 0.622; power < 0.20), with insufficient power to rule out meaningful gender differences in sexual activity rates (Table 2). Women had a mean QS-F score of 64.8 ± 18.5, while men reported a significantly higher mean QS-M score of 77.9 ± 14.2, with a high statistical power (p = 0.002; power > 0.90). The high power supports the occurrence of this gender difference finding. Among women, 20.0% were classified as having good-to-excellent sexual function, 37.5% as regular to good, 35.0% as unfavorable to regular, and 7.5% as null or unfavorable. In contrast, 43.3% of men were very satisfied, 10.0% moderately satisfied, and 46.7% partially satisfied, with no men reporting dissatisfaction. These distributions showed a statistically significant difference between sexes in the highest satisfaction category (p = 0.021), but the power of associations was very low for some comparisons (< 0.20). The low power undermines confidence in this categorical comparison despite statistical significance.

**Table 2:** Sexual activity and sexual function of men and women with atopic dermatitis (QS-F and QS-M scores).

Statistics and Categories	Women (n = 40)	Men (n = 30)	p-value (type I error)	Statistical power (1- type II error)
Reported Sexual activity: n (%)				
QS-F and QS-M: Mean score (± SD)	30 (75.0)	24 (80.0)	0.622	0.038
QS-F and QS-M: (categorical): n (%) (scores)	64.8 ± 18.5	77.9 ± 14.2	0.002	0.918
- Good to excellent (women) / Very satisfied (men) (82 to 100)	8 (20.0)	13 (43.3)	0.021	0.453
- Regular to good (women) / Moderately satisfied (men) (62 to 81)	15 (37.5)	3 (10.0)		0.661
- Unfavorable to regular (women) / Partially satisfied (men): n (%) (42 to 61)	14 (35.0)	14 (46.7)		0.112
- Null or unfavorable (women) / very unsatisfied/unsatisfied (men): n (%) (< 42)	3 (7.5)	0 (0.0)		0.115

SD, Standard Deviation; QS-F, Female Sexual Quotient; QS-M, Male Sexual Quotient.

Correlation analysis revealed that in women, higher QS-F scores were significantly associated with higher EQ-5D-3L index scores (*r* = 0.453, p = 0.003; power = 0.74), indicating that better sexual function was related to better perceived health. The moderate-to-high power (74%) supports this as a possible robust, clinically meaningful finding. Additionally, SCORAD scores correlated negatively with QS-F scores (*r* = -0.408, p = 0.008; power = 0.61), suggesting that increased disease severity was linked to reduced sexual satisfaction, but the power was only moderately high. In men, SCORAD was negatively associated with the satisfaction domain of the QS-M (*r* = -0.376, p = 0.041; power = 0.058), although no significant correlation was observed between total QS-M scores and EQ-5D-3L (power < 0.20). The very low power severely limits the interpretability of male findings.

These results highlight a psychosexual burden in patients with AD, particularly among women, whose sexual satisfaction was markedly lower than that reported in the general Brazilian population. The association between sexual function and overall health perception suggests that incorporating validated instruments such as the Sexual Quotients and EQ-5D-3L can enhance clinical understanding of disease impact.^10^ The gender-specific patterns observed further support the need for tailored interventions in the management of AD.

It should be noted that some nonsignificant comparisons were accompanied by very low statistical power (< 20%), mostly due to small sample size, which limits the ability to draw firm conclusions and indicates a higher likelihood of type II error. Therefore, these results should be interpreted with caution.

Another important limitation of the present study is concerned to its external validity. The study was conducted in a single university hospital; hence, the findings are to be considered preliminary, and they would benefit from replication in larger and more diverse samples.

In conclusion, AD exerts a measurable impact on sexual function and health-related quality of life, with more pronounced effects in women. These results reinforce the importance of integrating sexual health assessments into routine dermatological care and adopting a gender-sensitive approach in both evaluation and treatment.

## ORCID ID

Aline Bressan: 0000-0002-3296-5232

Natalia Troncoso: 0009-0005-3545-1498

Carla Jorge Machado: 0000-0002-6871-0709

Rita Fernanda Cortez de Almeida: 0000-0001-7904-998X

Sueli Carneiro: 0000-0001-7515-2365

## Research data availability

The entire dataset supporting the results of this study was published in this article.

## Financial support

None declared.

## Authors' contributions

Aline Bressan: The study concept and design; data collection, analysis and interpretation; writing of the manuscript or critical review of important intellectual content; final approval of the final version of the manuscript.

Natalia Troncoso: Critical review of important intellectual content; final approval of the final version of the manuscript.

Carla Jorge Machado: Data collection, analysis and interpretation; critical review of important intellectual content; final approval of the final version of the manuscript.

Rita Fernanda Cortez de Almeida: Critical review of important intellectual content; final approval of the final version of the manuscript.

Sueli Carneiro: Effective participation in the research guidance; critical review of important intellectual content; final approval of the final version of the manuscript.

## Conflicts of interest

None declared.
